# Endostatin Genetically Engineered Placental Mesenchymal Stromal Cells Carrying Doxorubicin-Loaded Mesoporous Silica Nanoparticles for Combined Chemo- and Antiangiogenic Therapy

**DOI:** 10.3390/pharmaceutics13020244

**Published:** 2021-02-10

**Authors:** Paz de la Torre, Juan L. Paris, Miguel Fernández-de la Torre, María Vallet-Regí, Ana I. Flores

**Affiliations:** 1 Grupo de Medicina Regenerativa, Instituto de Investigación Sanitaria Hospital 12 de Octubre (imas12), Avda. Cordoba s/n 28041, 28041 Madrid, Spain; torre-merino.paz@h12o.es; 2 Departamento de Química en Ciencias Farmacéuticas (Unidad Docente de Química Inorgánica y Bioinorgánica), Facultad de Farmacia, Universidad Complutense de Madrid, Instituto de Investigación Sanitaria Hospital 12 de Octubre (imas12), 28040 Madrid, Spain; 3 Centro de Investigación Biomédica en Red de Bioingeniería, Biomateriales y Nanomedicina (CIBER-BBN), 28029 Madrid, Spain; 4 Grupo de Enfermedades Raras, Mitocondriales y Neuromusculares, Instituto de Investigación Sanitaria Hospital 12 de Octubre (imas12), Avda. Cordoba s/n 28041, 28041 Madrid, Spain; miquel.fnandezt.imas12@h12o.es

**Keywords:** mesenchymal stromal cells, mesoporous silica nanoparticles, combination therapy, antiangiogenic therapy

## Abstract

Combination therapies constitute a powerful tool for cancer treatment. By combining drugs with different mechanisms of action, the limitations of each individual agent can be overcome, while increasing therapeutic benefit. Here, we propose employing tumor-migrating decidua-derived mesenchymal stromal cells as therapeutic agents combining antiangiogenic therapy and chemotherapy. First, a plasmid encoding the antiangiogenic protein endostatin was transfected into these cells by nucleofection, confirming its expression by ELISA and its biological effect in an ex ovo chick embryo model. Second, doxorubicin-loaded mesoporous silica nanoparticles were introduced into the cells, which would act as vehicles for the drug being released. The effect of the drug was evaluated in a coculture in vitro model with mammary cancer cells. Third, the combination of endostatin transfection and doxorubicin-nanoparticle loading was carried out with the decidua mesenchymal stromal cells. This final cell platform was shown to retain its tumor-migration capacity in vitro, and the combined in vitro therapeutic efficacy was confirmed through a 3D spheroid coculture model using both cancer and endothelial cells. The results presented here show great potential for the development of combination therapies based on genetically-engineered cells that can simultaneously act as cellular vehicles for drug-loaded nanoparticles.

## 1. Introduction

Cancer is the second leading cause of death worldwide. Although some people with cancer will have only one treatment, most people will receive a combination of treatments. Combination therapies hold great promise for cancer therapy [1]. The association of different therapeutic strategies allows exploiting the benefits of each modality while decreasing the limitations of each one. Antiangiogenic therapy has shown potential as a strategy for cancer treatment through starvation of cancer cells by preventing the formation of new blood vessels needed to feed the growing tumor. Within antiangiogenic drugs, endostatin is especially interesting because of its broad spectrum of action, having shown to be effective in over 65 different types of tumors [2,3]. Endostatin is a 20 kDa internal fragment of collagen XVIII, and its interaction with integrins involved in angiogenesis appears to be key for its action [4]. It is also capable of inducing apoptosis of endothelial cells [5]. However, antiangiogenic therapy is prone to the appearance of resistance and combining it with other therapeutic modalities with a different mechanism of action could reduce this limitation [6,7]. The most common strategy in that regard is combining antiangiogenic therapy with chemotherapy based on cytotoxic drugs [8]. One common chemotherapeutic drug that has been used in combination with antiangiogenic agents is doxorubicin (DOX) [9]. DOX is an anthracycline drug active against a broad range of cancers [10]. The significant off-target toxicity of traditional chemotherapy agents (such as cardiotoxicity in the case of DOX) has drawn much interest for targeted therapies, where the chemotherapeutic agent is selectively delivered to tumors, decreasing systemic toxicity [11]. One common way to achieve selective delivery of chemotherapeutic drugs to tumors is by the use of nanocarriers (cancer nanomedicine) [12]. These nanocarriers can accumulate in tumors either by passive or active targeting [13]. However, it has been recently highlighted that the efficiency of this tumor accumulation is quite low, and alternative strategies are necessary [14]. Actively-migrating cell vehicles can be used to transport drugs or nanoparticles towards tumors as a potential alternative [15]. Among the different types of cells that have been proposed as cancer-targeting agents (such as red blood cells, macrophages, mesenchymal stromal cells (MSC) or T cells), MSC appear especially promising cellular vehicles due to their capacity to selectively and actively migrate to tumors, in addition to their safety profile, when compared to other cell types such as macrophages, for example [16,17,18,19,20]. Using this strategy, true active targeting of nanoparticles for cancer therapy can be achieved [21]. MSC from the decidua (DMSC) of human placenta have been previously shown to migrate towards tumors in vitro and in vivo, and they even affect tumor growth by themselves [22,23]. We have previously shown that DMSC can be used to deliver different types of mesoporous silica nanoparticles (MSNs) towards mammary tumors both in vitro and in vivo [23,24,25]. Mesoporous silica nanoparticles present several advantages that make them very promising for drug delivery applications, such as their physicochemical stability, large surface area enabling a high drug loading capacity, good biocompatibility, easy synthesis and chemical modification [26,27,28,29]. Here, we propose the preparation of genetically-engineered DMSC producing endostatin and simultaneously carrying MSNs loaded with DOX for combined chemo- and antiangiogenic therapy. The effects of this combined strategy on a 3D multicellular spheroid model are evaluated.

## 2. Materials and Methods

### 2.1. Cell Lines and Reagents

Human umbilical vein endothelial cells (HUVECs) were obtained from Sigma-Aldrich (Madrid, Spain) and *N*-methyl-*N*-nitrosourea-induced rat mammary cancer cells (NMU cells) were purchased to the American Type Culture Collection (AATC; Manassas, VA, USA). Expansion media for NMU cells was purchased from Gibco (Thermo Fisher Scientific, Madrid, Spain) and media for HUVEC was from Innoprot (Bizkaia, Spain).

The reagents for isolation and expansion of decidua mesenchymal stromal cells (DMSC) were obtained from Gibco (Thermo Fisher Scientific, Madrid, Spain) except for fetal bovine serum (FBS) which was from Biowest (Labclinics, Madrid, Spain) and epidermal growth factor (EGF) from Sigma-Aldrich (Madrid, Spain).

The following reagents used for the synthesis of MSNs were obtained from Sigma-Aldrich (Madrid, Spain) and were used without further purification: Cetyltrimethylammonium bromide (CTAB), NaOH, tetraethyl orthosilicate (TEOS), (3-Aminopropyl) triethoxysilane (APTES), NH_4_NO_3_, rhodamine B isothiocyanate (RBITC). Doxorubicin hydrochloride was purchased from abcr GmbH (Karlsruhe, Germany).

### 2.2. Isolation and Culture of DMSC

Human placentas from healthy mothers were obtained from the Department of Obstetrics and Gynecology under written informed consent approved by the Ethics Committee from Hospital Universitario 12 de Octubre. To obtain DMSC, processing of placental membranes and culture of primary cells was done as previously described [30]. Briefly, placental membranes were digested by trypsin-EDTA and isolated cells were grown in complete medium consisting in Dulbecco’s modified Eagle’s medium (DMEM) supplemented with 2 mM of glutamine, 0.1 mM of sodium pyruvate, 55 mM β-mercaptoethanol, 10 mM non-essential amino acids, 100 units/mL penicillin/100 µg/mL streptomycin, 10% FBS and 10 ng/mL of EGF, at 37 °C, 5% CO_2_ and 95% humidity. Non-adherent cells were discarded after 5 d. The morphology, phenotype, maternal origin and mesenchymal characteristics of DMSC were reported in our previous study. At confluence, adherent cells were passaged and seeded at a density of 10^4^ cells per cm^2^.

### 2.3. Transfection of DMSC 

Plasmids containing the open reading frames of the endostatin gene or of the green fluorescent protein (GFP) gene (GeneCopoeia, Rockville, MD, USA) were delivered into DMSC by nucleofection, an electroporation-based transfection method, using the Nucleofector Technology and specific solutions from Lonza (Pontevedra, Spain). Briefly 3 × 10^5^ DMSC were resuspended in 100 μL of the mesenchymal cell specific solution, 1 μg of plasmid was added, and were transferred to a sterile 0.2-cm cuvette to be electroporated using the U-34 protocol of the nucleofector.

Transfection with the GFP plasmid was quantitatively evaluated by flow cytometry (FACS). After nucleofection, transfected DMSC were cultured for 48 h using fresh complete culture medium. Cells were then collected by trypsinization and centrifugation, and resuspended in phosphate buffered saline (PBS). The fluorescence intensity of 10,000 cells was quantified by FACS in a BD FACSCalibur system.

Transfection of the endostatin plasmid was evaluated by ELISA using a commercial human endostatin ELISA Kit (Abcam, Cambridge, UK). Endostatin concentration in the culture media of transfected and non-transfected DMSC was determined 24 and 48 h after nucleofection following the manufacturer’s instructions.

### 2.4. Chick Embryo Model

The biological effect of the produced endostatin was assessed by a chorioallantoic membrane (CAM) assay in ex ovo grown chick embryos [31].

The model was performed by incubating fertilized eggs from embryonic day (ED)-1 to ED-4 in a hatcher with rotation at 38 °C at 60% humidity. On ED-4, the embryos were de-shelled following an established method [32] and transferred into sterilized weighing boats covered with a sterile square Petri dish. The embryos were then transferred to a static humidified incubator at 38 °C, 60% humidity and 0.5% CO_2_. On ED-7, culture media from transfected and non-transfected DMSC (48 h after electroporation) were employed to soak cellulose discs that were placed on top of the CAM of the chick embryos. PBS was employed as a control. The evolution of the surrounding vasculature was evaluated by stereomicroscope and photographed using a 10× objective for the following 48 h. Since chick embryos were sacrificed before potential hatching, and experiments were carried out according to available ethical guidelines, no especial institutional approval was necessary (as confirmed by the Animal Housing Service from UCM).

### 2.5. Preparation of MSNs

MSNs were synthesized as previously described [23]. Briefly, 1 g of CTAB was dissolved in deionized (DI) water (480 mL) and 2 M NaOH (3.5 mL) were added with magnetic stirring. The mixture was heated to 80 °C and a mixture of TEOS (4.5 mL) and APTES (0.5 mL) was slowly added during 20 min. The particles were then stirred at 80 °C for 2 h. The resulting particles were collected by centrifugation and washed with water and ethanol. CTAB extraction was performed at 75 °C overnight by ion exchange with an NH_4_NO_3_ solution in 95% EtOH (10 mg/mL). The particles were then centrifuged and washed with water and ethanol. To prepare rhodamine B-labeled MSNs, 1 mg of RBITC was reacted with 2.2 µL of APTES in 100 µL of ethanol. After stirring for 2 h at room temperature, it was combined with the mixture of TEOS and APTES and added to the CTAB solution as previously described.

To prepare DOX-loaded MSNs (DOX@MSN), 20 mg of MSNs were dispersed in 8 mL of a DOX solution in PBS (1 mg/mL of drug). After stirring at room temperature overnight in the dark, the particles were thoroughly washed with PBS to remove non-loaded drug. To study DOX release from DOX@MSN, a suspension of the particles in PBS was incubated at 37 °C with stirring for 7 d. At predetermined time points (3 h, 1 d, 2 d, 5 d, 6 d and 7 d), an aliquot of the suspension was centrifuged and DOX fluorescence in supernatant was determined with a plate reader (Enspire, PerkinElmer, Waltham, MA, USA).

### 2.6. Nanoparticle Characterization

The MSNs were characterized by small angle X-ray diffraction (SAXRD) in a Philips X-Pert MPD diffractometer equipped with Cu Kα radiation. Fourier transformed infrared (FTIR) spectra were obtained in a Nicolet (Thermo Fisher Scientific, Madrid, Spain) Nexus spectrometer equipped with a Smart Golden Gate attenuated total reflectance (ATR) accessory. Surface morphology was analyzed by scanning electron microscopy (SEM) in a JEOL 6400 electron microscope. N_2_ adsorption was carried out on a micromeritics ASAP 2010 instrument; surface area was obtained by applying the Brunauer, Emmet and Teller (BET) method to the isotherm and the pore size distribution was determined by the Barrett, Joyner and Halenda (BJH) method from the desorption branch of the isotherm. The mesopore size was determined from the maximum the pore size distribution curve. The Z-potential and hydrodynamic size of nanoparticles were measured in deionized water by means of a Zetasizer Nano ZS (Malvern Instruments) equipped with a 633 nm ‘‘red” laser.

### 2.7. Incubation of DMSC with MSNs

DMSC incubation with MSNs was performed following a previously developed protocol [23]. DMSC were plated at a density of 10^4^ cells per cm^2^ 24 h before incubation with MSNs. Then, the culture medium was removed and replaced with 200 µg/mL MSNs in serum-free DMEM for 2 h. Afterwards, the medium was removed, and the cells were washed 3 times with PBS before adding fresh complete culture medium.

Cell uptake of rhodamine-labeled MSNs was evaluated by fluorescence microscopy in an Evos FL Cell Imaging System equipped with three LED light cubes (λ_EX_ (nm); λ_EM_ (nm)): DAPI (357/44; 447/60), GFP (470/22; 525/50) and RFP (531/40; 593/40) from AMG (Advance Microscopy Group). DAPI (4’,6-diamidino-2-phenylindole) at 1 µg/mL was used to stain and visualize the nuclei.

### 2.8. Coculture of Mammary NMU Cancer Cells and DMSC Carrying DOX@MSN

DMSC carrying DOX@MSN were cocultured with NMU cells. NMU cells were seeded in 24 well plates at a density of 20,000 cells per well. After 24 h, DMSC with or without DOX@MSN were seeded in Transwell culture inserts (0.4 µm pore, polycarbonate membranes, tissue cultured treated from Costar, Thermo Fisher Scientific, Madrid, Spain) in a ratio 1:2 (DMSC:NMU). After 24 and 48 h, NMU cell viability was evaluated by Alamar Blue assay (Invitrogen, Thermo Fisher Scientific, Madrid, Spain) following the manufacturer’s instructions. At the 48 h timepoint, DMSC viability was also assessed by the same method. NMU cell viability was expressed as a percentage with respect to the viability of a culture of NMU cells without coculture with DMSC.

### 2.9. Design of the Final Cell Platform: Endostatin Transfected and MSN Loaded DMSC

To test the feasibility of combining electroporation-mediated transfection and nanoparticle loading, DMSC were transfected with the GFP plasmid by nucleofection as described in the Section 2.3. Transfected cells were plated and 24 h later, incubated with 200 µg/mL rhodamine-labeled MSNs for 2 h in serum-free DMEM. After washing non-internalized nanoparticles, DMSC were incubated in complete medium and the presence of both GFP expression and nanoparticle uptake was evaluated by fluorescence microscopy 24 h later.

The designed final cell platform consisting of endostatin-expressing DMSC carrying DOX@MSNs, was made following this tested protocol, employing the endostatin plasmid for transfection instead of the GFP one, and incubating the cells with DOX@MSN.

### 2.10. In Vitro Migration of DMSC

The in vitro migration capacity of this final cell platform towards NMU cancer cells was evaluated as previously described [23,24]. Non-modified DMSC were used as positive control, and migration in the absence of NMU coculture was used as a negative control. Briefly, NMU cells were seeded in 24 well plates 24 h in advance, as was described for the coculture experiments. In this case, non-modified DMSC or the final cell-platform were seeded in Millicell culture plate inserts with 8 μm pore polycarbonate membranes (Merck Millipore, Spain). Furthermore, 3 × 10^5^ DMSC in 300 µL of serum-free DMEM were seeded in the inserts which were then placed over the wells containing the NMU cells. Migration was assessed at 24 h by the CytoSelect 24-Well Cell Migration Assay (Cell Biolabs, Bionova Cientifica, S.L., Madrid, Spain). Non-migrant cells were removed from the top of the membrane and migrant cells on the bottom of the polycarbonate membrane were stained with the cell dye solution according to the manufacturer’s instructions. Color of the stained cells was extracted and absorbance at 560 nm was quantified with the plate reader Enspire (PerkinElmer). All experiments were done in triplicate.

### 2.11. Conditioned Media Obtaining

Conditioned medium was recovered from 4 experimental conditions: untreated DMSC, endostatin-transfected DMSC, DOX@MSN-loaded DMSC and the final cell platform (endostatin-transfected and DOX@MSN-loaded DMSC, see Section 2.9). First, DMSC were distributed in 4 nucleofection cuvettes at 3 × 10^5^ cells each. Electric shock was applied to all of them but only two cuvettes contained the endostatin plasmid. Cells from each cuvette were seeded in a 35 mm well in 1.2 mL of complete medium, and 24 h later, media from one untreated DMSC and one DMSC plus endostatin wells were collected and preserved before adding the DOX@MSN to the cells in serum-free medium, as described in Section 2.9. After washing the nanoparticles, these two wells were refilled with the preserved medium, and 24 h later, the media from the four wells were independently recovered and spun down at 10,000 rpm for 10 min at 4 °C to eliminate cell debris. Supernatant is the conditioned media and was aliquoted and stored at −80 °C until use.

### 2.12. Multicellular 3D Tumor Spheroid Formation

A multicellular 3D tumor spheroid model containing NMU cells and HUVEC was performed to evaluate the in vitro therapeutic efficacy of the final cell platform. The formation of the spheroids was carried out culturing the cells in U-shaped-bottom wells (NUNC, Fisher Scientific, Madrid, Spain). In a first step, 10^4^ NMU cells were seeded onto the bottom of the plate. Spheroids were growing for 5 days, and partial media changes were carried out every 2–3 days. At day 5, 10^4^ PKH26-stained HUVEC were added on top of each NMU spheroid. PKH26 is a lipophilic red-fluorescent dye for cell membrane labelling (Sigma-Aldrich, Spain) and was used following manufacturer instructions. After 5 days of coculture, NMU-HUVEC spheroids were ready to be treated with the conditioned media from DMSC exposed to the different experimental conditions. Viability of cells in spheroids was evaluated 24 h later by Alamar Blue assay (Invitrogen, Thermo Fisher Scientific, Madrid, Spain) following manufacturer instructions.

### 2.13. Apoptosis Detection

The apoptotic effect produced by the conditioned media on the spheroids and on monolayer cultures was evaluated after 48 h of exposure. Spheroids were fixed in 3% neutral formalin overnight. After two PBS washes apoptosis was detected using CellEvent Caspase 3/7 apoptosis marker (Invitrogen, Madrid, Spain) according manufacturer instructions.

Spheroids were visualized with a Zeiss LSM 510 meta inverted confocal microscope (Carl Zeiss Meditec Iberia SAU, Madrid, Spain). In order to analyze the cells throughout the 3D structure, images were collected from different depths within the spheroid acquiring a stack of confocal images in the z plane.

Monolayer cultures were fixed in 10% neutral formalin for 15 min, twice PBS washed, stained using CellEvent Caspase 3/7 apoptosis marker and nuclei counterstained with 20 ng/mL of PureBlu Hoechst 33,342 nuclear staining dye from Bio Rad (Madrid, Spain). Images were captured by Zoe Fluorescent Cell Imager from Bio Rad (Madrid, Spain).

### 2.14. Statistical Analysis

Statistical analyses were performed by one-way ANOVA using GraphPad Prism Software version 9.0.0 (GraphPad Software, San Diego, CA, USA). Statistical significance was defined for *p* values below 0.05 (after correction for multiple comparisons).

## 3. Results and Discussion

### 3.1. Genetic Engineering of DMSC to Express Endostatin

**Figure 1:**
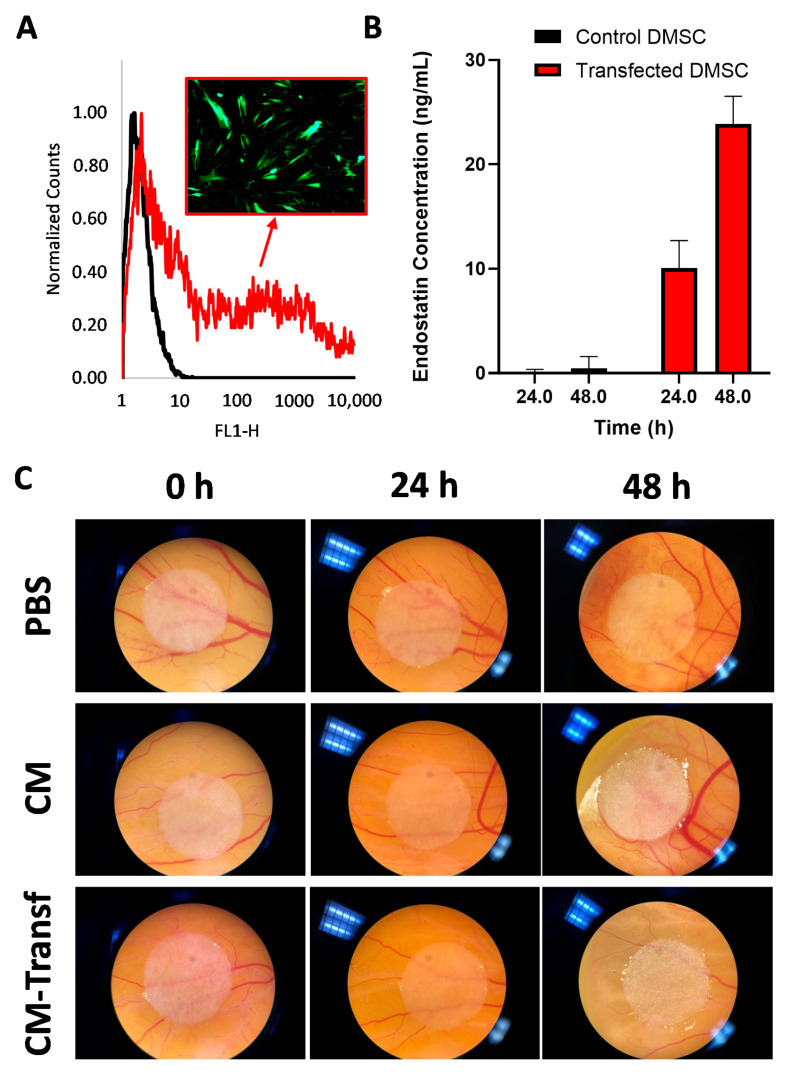
Flow cytometry histograms of DMSC without (black) and with (red) transfection with a green fluorescent protein (GFP) plasmid through electroporation and insert with a fluorescence microscopy image of GFP-transfected DMSC (**A**); ELISA measurement of endostatin in the culture medium of DMSC and transfected DMSC with the endostatin plasmid (**B**); and the effect on chick chorioallantoic membrane (CAM) vasculature of PBS and conditioned media of DMSC (CM) and transfected DMSC (CM-Transf) with the endostatin plasmid (**C**). Data are means ± SD; *n* = 3.

Nucleofection protocol for plasmid transfection of DMSC was first developed using a GFP-encoding plasmid. Under the tested conditions, 56.1 ± 0.4% of the treated DMSC expressed GFP as determined by flow cytometry (Figure 1A). These optimized conditions were used to transfect DMSC with the plasmid encoding endostatin. Figure 1B shows the amount of endostatin released to the culture media 24 and 48 h after transfection, as measured by ELISA. While no endostatin could be detected in the media of control cells, endostatin was detected in the culture media of DMSC at both time points after transfection. The concentration of endostatin measured in DMSC culture media was larger than those reported in the literature for other mesenchymal stromal cells transfected by non-viral methods [33]. Since endostatin concentration in the culture medium of transfected cells was 2.37 times larger at 48 h than at 24 h, this time point was selected for further experiments. To test the antiangiogenic efficacy of the endostatin-producing DMSC, a functional anti-vascular assay in the CAM of chick embryos was performed. CAM has been chosen as the model to evaluate the effect of endostatin on the angiogenesis process, given the provided advantages respect to other methods, as the fast vascular growth, the possibility of direct monitoring, or the absence of requirement for animal protocol approval, among others [34]. Aliquots of the culture media of transfected DMSC were used to soak cellulose discs that were then placed on top of the CAM of chick embryos grown ex ovo. Two days after exposure, blood vessels in the area surrounding the discs were reduced compared to the vessels in its starting point, whereas control samples exposed to PBS or culture media from non-transfected cells showed vascular growth when compared to their initial time point (Figure 1C). We performed this qualitative evaluation based on the assessment of the vascular morphology and density, as the employed model did not enable an easy quantification [35].

It is worth noting here that the role of MSCs in angiogenesis is a controversial issue, since depending on the experimental conditions and origin of the cells, both angiogenic and antiangiogenic effects have been described [36]. Regardless of their inherent properties, MSCs engineered to express antiangiogenic factors have been shown to present good potential for the development of anticancer therapeutics [37,38].

### 3.2. Mesoporous Silica Nanoparticles (MSNs) Loading into DMSC

**Figure 2:**
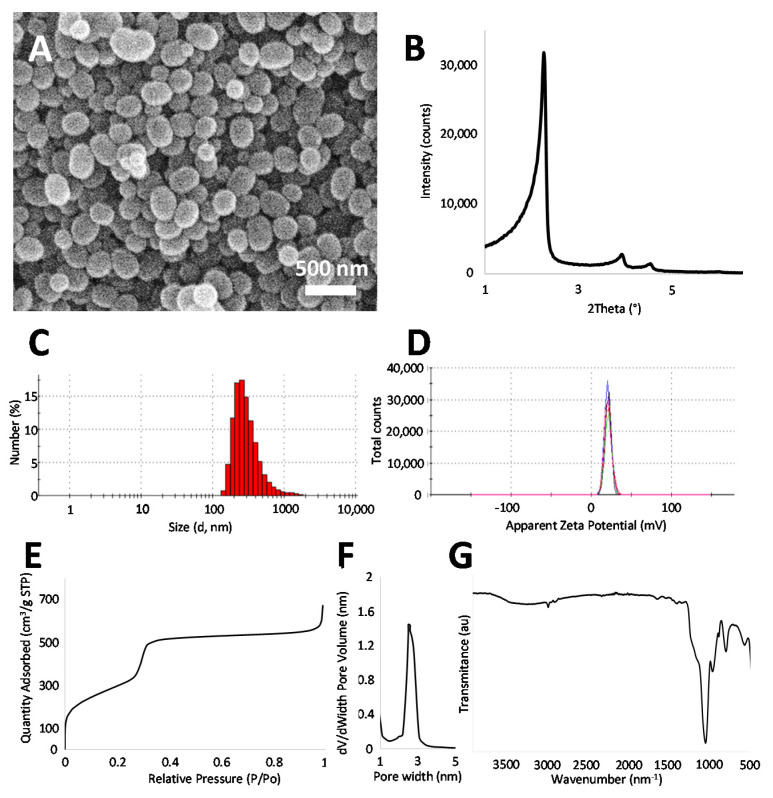
Characterization of the prepared MSNs by different techniques. SEM micrograph showing particle size and morphology (**A**); SAXRD pattern with characteristic maxima of MCM-41 type materials, confirming the ordered porosity (**B**); size distribution by DLS (**C**); Z-potential (**D**); N_2_ adsorption results showing a type IV N_2_ adsorption isotherm without hysteresis, typical of MCM-41 materials (**E**) and mesopore size distribution (**F**); FTIR spectrum showing the expected bands for surfactant-extracted mesoporous silica(**G**).

Amino-functionalized MSNs were synthesized and characterized (Figure 2). The SEM images show round-shaped particles of 206.35 ± 45.25 nm in diameter (Figure 2A). The size (243.4 ± 20.09 nm) and positive charge of the prepared MSNs was further confirmed by dynamic light scattering (DLS) and Z-potential measurements, respectively (Figure 2C,D). Small angle X-ray diffraction shows a typical pattern of pore order associated with MCM-41 type mesoporous silica (Figure 2B) [39]. The porosity of the prepared MSNs was further confirmed by N_2_ adsorption (Figure 2E,F), which shows typical large values of surface area (1094.7 m^2^/g) and a pore size of 2.6 nm. Finally, FTIR (Figure 2G) also shows a typical spectrum for surfactant-extracted amino-functionalized silica nanoparticles [23]. After DOX loading, a characteristic red color in the particles confirms the presence of the drug in the formulation (Figure S1). These particles presented an initial burst release (26.82 ± 1.74% drug released at 3 h) followed by a sustained DOX release at 37 °C in PBS, with 50.81 ± 5.11% of drug released within 7 d (Figure S1). These values are in good agreement with those for similar DOX release MSNs reported in the literature [40,41,42].

**Figure 3:**
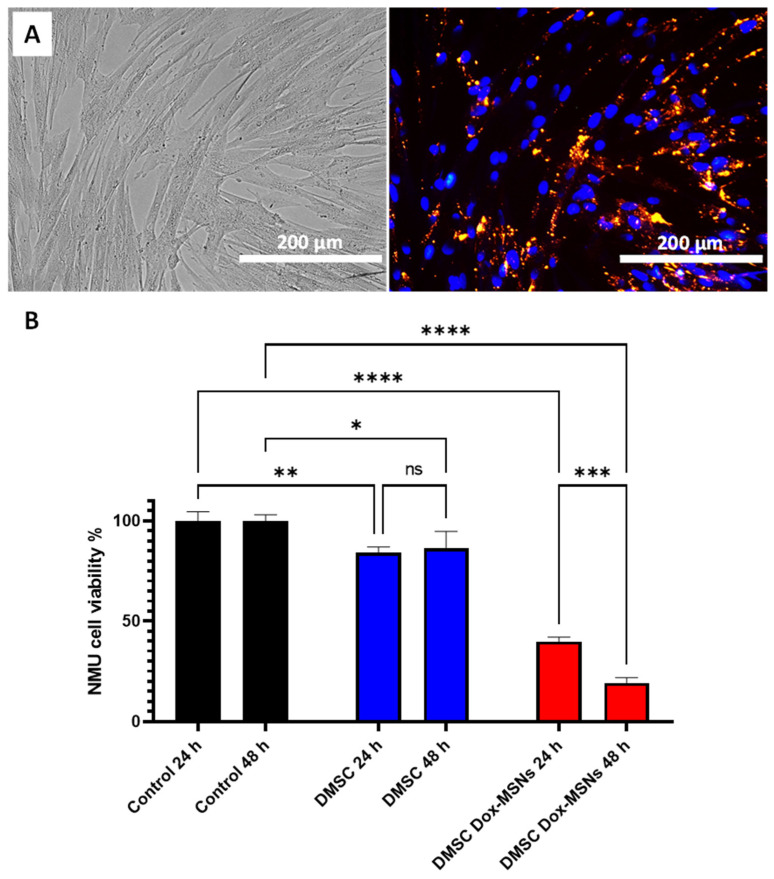
Microscopy of DMSC cultured with red-fluorescent (R)-MSNs showing bright field (left) and fluorescence images (right). Nuclei are stained in blue and R-MSNs are fluorescent in the red channel (**A**); NMU cell viability in coculture with DMSC without and with DOX@MSNs (**B**). Data are means ± SD, *n* = 3; ns *p* > 0.05; * *p* ≤ 0.05; ** *p* ≤ 0.01; *** *p* ≤ 0.001; **** *p* ≤ 0.0001.

Positively-charged MSNs were selected for this study since we had previously observed that they would be efficiently internalized in DMSC [23]. Red-fluorescent MSNs (R-MSNs) were incubated with DMSC in serum-free medium to evaluate nanoparticle uptake by DMSC. The fluorescence microscopy images (Figure 3A) show successful uptake of positively charged MSNs by DMSC, since almost all of the cells present red fluorescence within their cytoplasm. This result is in good agreement with our previous results [23] using these types of nanoparticles and cells. Doxorubicin-loaded MSNs (DOX@MSNs) were then delivered into DMSC, and a coculture experiment between DMSC and NMU cancer cells, through a Transwell insert, was carried out to test the cytotoxic effect of drug-releasing nanoparticle-containing DMSC. Coculture of NMU cells with DMSC without any treatment certainly already produced a significant reduction on NMU cell viability (Figure 3B) as it had been previously reported [22,25]. However, when the DMSC were carrying DOX@MSNs, NMU cell viability was so drastically reduced that only 19.32 ± 2.6% of tumor cells were viable after 48 h of coculture. At this same time point, DMSC viability was also drastically affected (Figure S2), with only 29.45 ± 1.06% viability. This loss of DMSC viability after a certain incubation time with the nanoparticles might be considered a positive feature of our setup, since the effect of long-term surviving MSC after administration might give rise to safety concerns [43]. However, this survival time of DMSC should be long enough to ensure their migration and accumulation in the target cancer tissue [22].

### 3.3. Combined MSN Loading and Gene Transfection of DMSC: Cell Platform

After having tested both, endostatin transfection and nanoparticle loading of DMSC separately, we planned to explore the conjugation of both therapeutic strategies in the same platform. In this way, the antiangiogenic effect of endostatin (produced by transfected cells) and the cytotoxic effect of doxorubicin (released from nanoparticles) would be combined to improve therapeutic efficacy.

**Figure 4:**
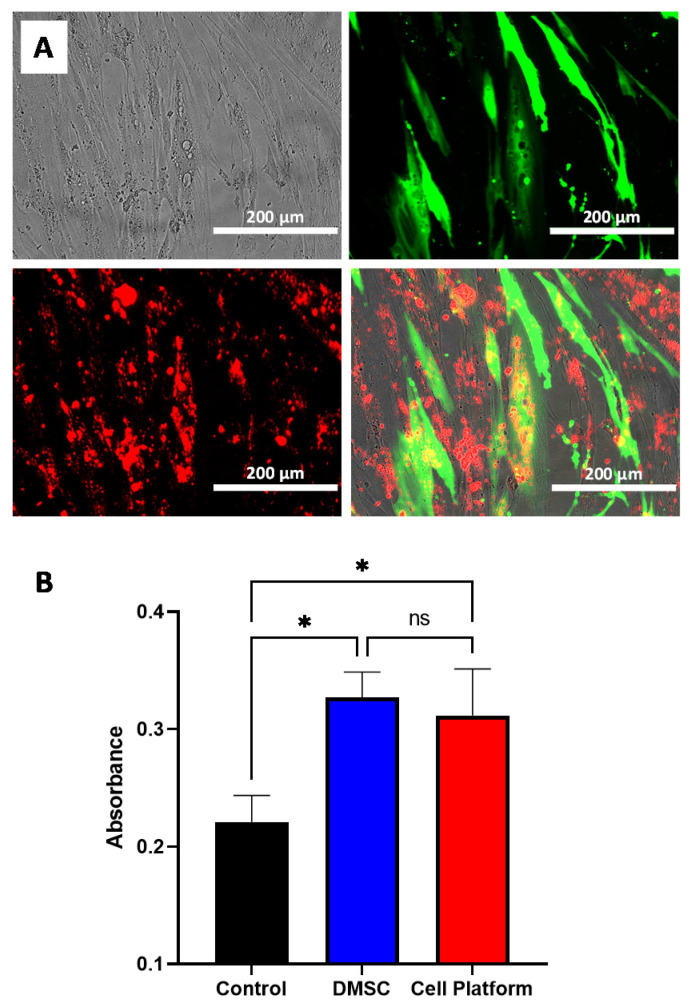
Bright field and fluorescence microscopy of GFP-transfected DMSC also cultured with R-MSNs. Green fluorescence due to GFP expression and R-MSNs fluorescence in the red channel (**A**); DMSC in vitro migration assay towards NMU culture without (DMSC) and with transfection plus DOX@MSNs (cell platform) (**B**). Data are means ± SD, *n* = 3; ns *p* > 0.05; * *p* ≤ 0.05.

Firstly, to check the strategy, we transfected DMSC with the GFP-encoding plasmid and two days later, R-MSNs were added to the culture. Fluorescence microscopy shows successful GFP transfection and nanoparticle uptake (Figure 4A) with most cells presenting red fluorescence and some cells showing both GFP expression and nanoparticle uptake.

One of the bases of the therapeutic use of MSC in cancer is their tumor tropism behavior [44]. It has been previously shown that DMSC migrate to tumors in vitro and in vivo [22,23]. To check whether DMSC maintained their migratory properties while carrying both therapeutic strategies, in other words, transfected with the therapeutic plasmid and simultaneously carrying DOX@MSNs, we carried out an in vitro migration assay against an NMU cancer cell culture employing a Transwell assay. The results obtained from this experiment show that the in vitro cell migration of the final cell platform (transfected cells carrying DOX@MSNs) towards cancer cell culture was equivalent to that of non-modified DMSC (Figure 4B). At the same time, cell migration of both groups was significantly larger than migration of a control of DMSC without cancer cells in the well below. This result shows that DMSC retain their in vitro migratory properties toward tumor cells after transfection and DOX@MSN uptake which provides a preliminary validation for further testing of our strategy.

**Figure 5:**
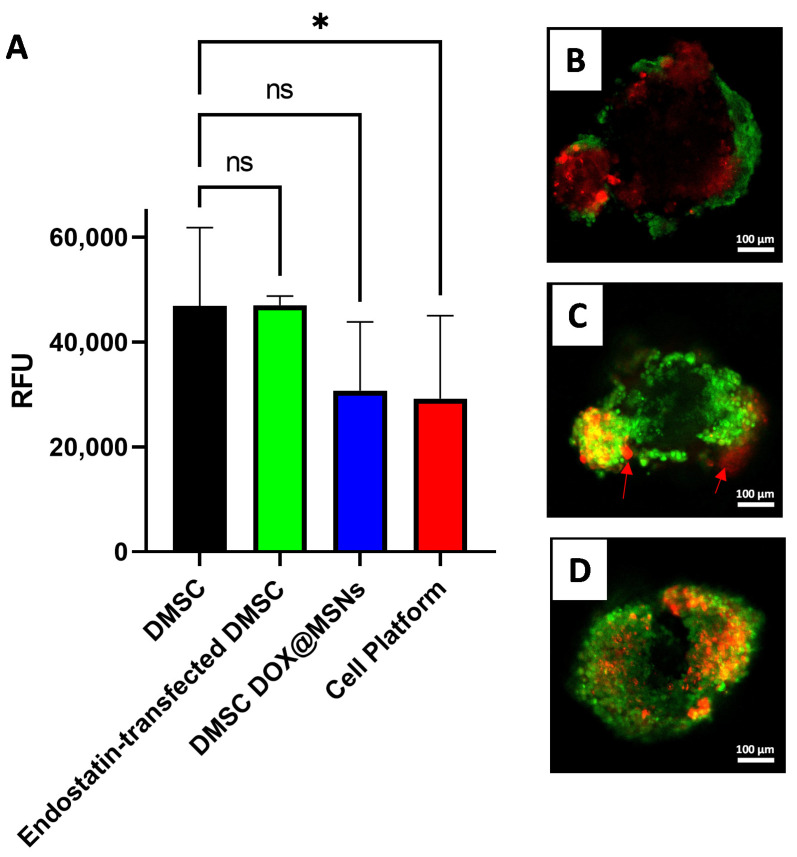
Metabolic activity measured by Alamar Blue assay (**A**) of multicellular 3D spheroids after incubation with conditioned media of DMSC from the different treatment groups. Confocal microscopy images of multicellular 3D spheroids at the end of the experiment, after incubation with conditioned media of DMSC (**B**); DMSC-Dox@MSNs (**C**) or the complete cell platform (**D**). Red fluorescence shows PKH26 membrane label of HUVEC cells, green fluorescence shows apoptotic cells labeled with CellEvent Caspase 3/7 apoptosis marker. Data are means ± SD, *n* = 8; ns *p* > 0.05; * *p* ≤ 0.05.

To test the in vitro efficacy of the combined therapeutic strategy (cell platform), we employed a multicellular 3D spheroid model formed by the coculture of tumoral and endothelial cells. It is considered that spheroids mimic tumor behavior more accurately than two dimensional (2D) cell cultures and represent a much more relevant model for evaluating anticancer therapies [45,46,47]. Our multicellular model may provide information about the effect of the developed cell platform on cancer and endothelial cells simultaneously. First, NMU cancer cells were left to form the spheroids and then PKH26-stained HUVEC cells were added. Conditioned media from DMSC cultures exposed to different treatments (including the complete cell platform) were added to the multicellular spheroids and their effect was evaluated by measuring cellular viability of the spheroids and the induction of apoptosis. In addition to the control (spheroids incubated with DMSC media without further treatment) and the cell platform treatment, conditioned media from endostatin-transfected DMSC and from DMSC DOX@MSNs loaded were also tested. Figure 5A shows that only the treatment with the combined strategy produced a significant decrease (after correction for multiple comparisons) in spheroids viability with regards to control treatment. Even so, as expected, the DMSC-DOX@MSNs treatment also led to loss of cellular viability in spheroids. For this reason, we decided to evaluate the apoptotic profile of the cells in the spheroids exposed to the different treatments. For this purpose, we selected CellEvent Caspase 3/7 as a fluorescent apoptosis indicator since it had already been shown to be useful for apoptosis detection within multicellular spheroids [48,49]. First, we checked the effect of incubation of monolayer cultures of either NMU (Figure S3) or HUVEC (Figure S4) cells with the conditioned media from the different DMSC treatments. Treatment with the conditioned media from DOX@MSN-loaded DMSC, confirmed the already reported sensitivity of NMU [23] and also of HUVEC cells [50,51], to the cytotoxic effects of doxorubicin. In addition, the treatment of monolayer cultures revealed a much more prominent effect of endostatin on HUVEC cells compared to NMU cells, both regarding cell density and apoptosis, and in consequence, the HUVEC monolayer was noticeably sensitive to the deleterious effects of the conditioned medium from the complete cell platform with an evident loss of cellular population. In spheroids, confocal microscopy revealed that the exposition to conditioned medium from control DMSC (without further treatment) produced some apoptosis, only visible on the surface of the spheroids (Figure 5B). This is not a surprising result since DMSC affects the viability of NMU cells as shown in Figure 3B and as reported elsewhere [22,25]. It is worth noting that there was virtually no colocalization of red fluorescence (from labeled HUVEC endothelial cells) and green fluorescence (from apoptotic cells), thus informing of the absence of any toxic effect of DMSC on HUVEC. For spheroids treated with conditioned media from the DMSC-DOX@MSNs group (Figure 5C), apoptosis was significantly increased with respect to spheroids treated with conditioned media from the DMSC group. In this case, apoptosis was no longer limited exclusively to the outer region of the spheroid but rather comprising both endothelial and tumor cells, in accordance with what was observed in the monolayer cultures. However, a significant portion of red-fluorescent HUVEC cells did not show green fluorescence indicative of apoptosis (arrows in Figure 5C). Finally, for the spheroids from the complete cell platform group (endostatin-transfected DMSC carrying DOX@MSNs) (Figure 5D), apoptosis was much more extensive and, more importantly, comprised all the regions where HUVEC cells were observed, with almost complete colocalization of green fluorescence wherever there was red fluorescence. These results indicate that the combined therapeutic strategy drastically affected 3D multicellular tumor spheroids, but also ensuring a profound effect in endothelial cells.

These results show the great potential of the designed cell platform for combined therapy in cancer. The concurrent use of two therapeutical approaches, the cytotoxic effect of doxorubicin and the antiangiogenic effect of endostatin, may offer several advantages in the treatment of solid tumors. Due to their vascular abnormality, tumors are known to have impaired blood perfusion and offer resistance to chemotherapy [7]. Endostatin has been used to normalize the aberrant vasculature in tumors [52] and this effect has even been explored in clinical settings [53]. In combination with doxorubicin, endostatin has been shown significant tumor and metastasis inhibition in mice 4T1 breast cancer [54]. Nevertheless, systemic administration of these drugs implies toxic effects. The use of DMSC as a cellular vehicle for anti-tumor drugs could reduce their unwanted side effects and could also increase therapeutic doses at the local tumor site [23,24,25]. In the future, validation of our cell platform strategy in different in vivo models will be necessary. In addition, incorporation of stimuli-responsive nanoparticles for a controlled drug release could further improve this antitumor strategy.

## Data Availability

Data is available from the authors upon request.

## Supplementary Materials

The following are available online at https://www.mdpi.com/1999-4923/13/2/244/s1, Figure S1: Images showing DOX presence in DOX@MSNs, release profile of DOX from DOX@MSNs in PBS; Figure S2: DMSC viability (without and with Dox-loaded MSNs) after 48 h in coculture with NMU cells determined by Alamar Blue assay; Figure S3: Fluorescence microscopy images of NMU cancer cells after incubation with conditioned media of DMSC from different treatment groups; Figure S4: Fluorescence microscopy images of HUVECs after incubation with conditioned media of DMSC from different treatment groups.
