# Supplementary material for: Endostatin Genetically Engineered Placental Mesenchymal Stromal Cells Carrying Doxorubicin-Loaded Mesoporous Silica Nanoparticles for Combined Chemo- and Antiangiogenic Therapy
Source: Pharmaceutics. 2021 Feb 10;13(2):244. doi: 10.3390/pharmaceutics13020244 (PMC7916487; doi:10.3390/pharmaceutics13020244)
Supplement: Supplementary file 1 [file pharmaceutics-13-00244-s001.pdf]

pharmaceutics

Supplementary Materials: Endostatin Genetically Engineered Placental Mesenchymal Stromal Cells Carrying Doxorubicin-Loaded Mesoporous Silica Nanoparticles for Combined Chemo- and Antiangiogenic Therapy

Paz de la Torre, Juan L. Paris , Miguel Fernández-de la Torre, María Vallet-Regí and Ana I. Flores

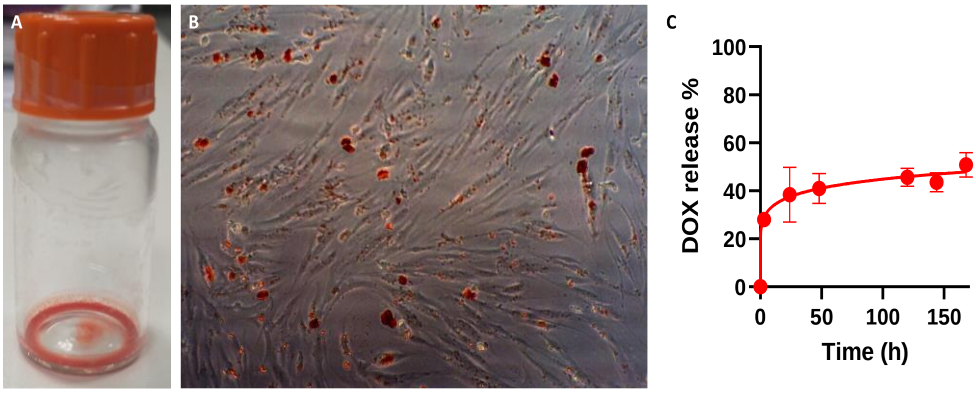

Figure S1. Photograph of dry DOX@MSNs (A) and microscopy transmission image of DOX@MSNs in culture with DMSC (B) showing a characteristic punctuated red color inside DMSC that indicates successful DOX loading. DOX release profile from DOX@MSNs in PBS at 37 °C under stirring (C).

Publisher's Note: MDPI stays neutral with regard to jurisdictional claims in published maps and institutional affiliations.

Copyright: © 2020 by the authors. Submitted for possible open access publication under the terms and conditions of the Creative Commons Attribution (CC BY) license (http://creativecommons.org/licenses/by/4.0/).

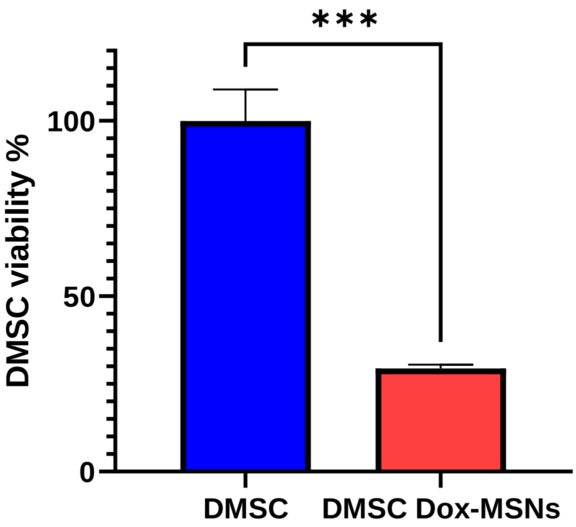

Figure S2. DMSC viability (without and with Dox-loaded MSNs) after 48 h in co-culture with NMU cells determined by Alamar Blue assay. Data are Means ±SD, n=3; ***p ≤ 0.001.

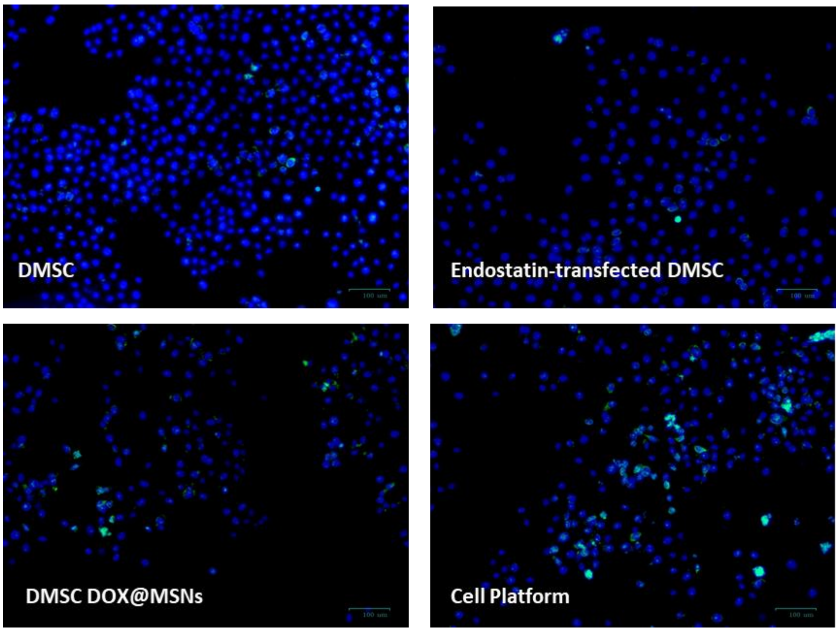

Figure S3. Fluorescence microscopy images of NMU cancer cells after incubation with conditioned media of DMSC from different treatment groups. Blue fluorescence corresponds to cell nuclei stained with Hoechst 33342. Green fluorescence shows apoptotic cells labeled with CellEvent Caspase 3/7 apoptosis marker.

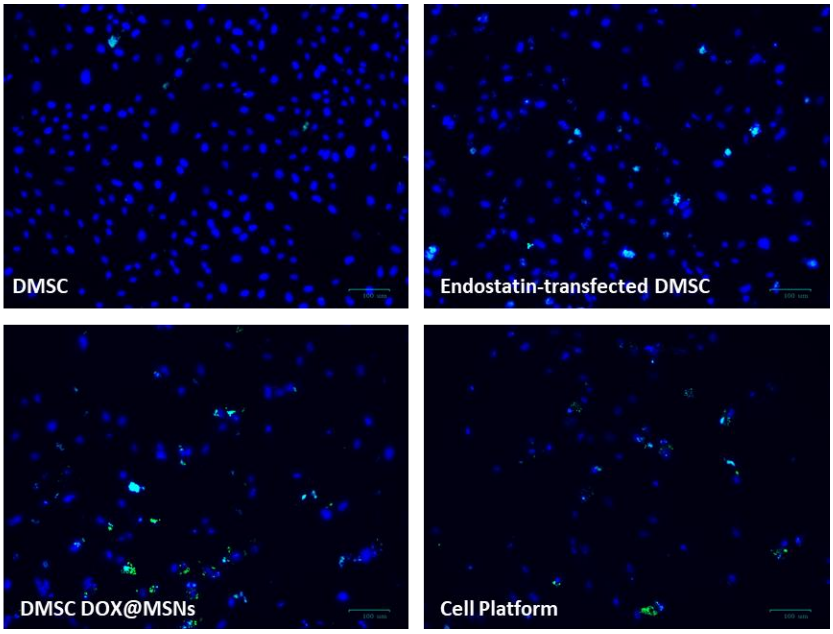

Figure S4. Fluorescence microscopy images of HUVEC endothelial cells after incubation with conditioned media of DMSC from different treatment groups. Blue fluorescence corresponds to cell nuclei stained with Hoechst 33342. Green fluorescence shows apoptotic cells labeled with CellEvent Caspase 3/7 apoptosis marker.
